# Determining clinical biomarkers to predict long-term SARS-CoV-2 antibody response among COVID-19 patients in Bangladesh

**DOI:** 10.3389/fmed.2023.1111037

**Published:** 2023-05-24

**Authors:** Tasnuva Ahmed, S. M. Tafsir Hasan, Afroza Akter, Imam Tauheed, Marjahan Akhtar, Sadia Isfat Ara Rahman, Taufiqur Rahman Bhuiyan, Tahmeed Ahmed, Firdausi Qadri, Fahima Chowdhury

**Affiliations:** ^1^Infectious Diseases Division, International Centre for Diarrhoeal Disease Research, Bangladesh, Dhaka, Bangladesh; ^2^Nutrition and Clinical Services Division, International Centre for Diarrhoeal Disease Research, Bangladesh, Dhaka, Bangladesh; ^3^Office of the Executive Director, International Centre for Diarrhoeal Disease Research, Bangladesh, Dhaka, Bangladesh

**Keywords:** SARS-CoV-2, biomarkers, antibody, prediction, Bangladesh, COVID-19, long-term immune response

## Abstract

**Background:**

Information on antibody responses following SARS-CoV-2 infection, including the magnitude and duration of responses, is limited. In this analysis, we aimed to identify clinical biomarkers that can predict long-term antibody responses following natural SARS-CoV-2 infection.

**Methodology:**

In this prospective study, we enrolled 100 COVID-19 patients between November 2020 and February 2021 and followed them for 6 months. The association of clinical laboratory parameters on enrollment, including lactate dehydrogenase (LDH), neutrophil–lymphocyte ratio (NLR), C-reactive protein (CRP), ferritin, procalcitonin (PCT), and D-dimer, with predicting the geometric mean (GM) concentration of SARS-CoV-2 receptor-binding domain (RBD)-specific IgG antibody at 3 and 6 months post-infection was assessed in multivariable linear regression models.

**Result:**

The mean ± SD age of patients in the cohort was 46.8 ± 14 years, and 58.8% were male. Data from 68 patients at 3 months follow-up and 55 patients at 6 months follow-up were analyzed. Over 90% of patients were seropositive against RBD-specific IgG till 6 months post-infection. At 3 months, for any 10% increase in absolute lymphocyte count and NLR, there was a 6.28% (95% CI: 9.68, −2.77) decrease and 4.93% (95% CI: 2.43, 7.50) increase, respectively, in GM of IgG concentration, while any 10% increase for LDH, CRP, ferritin, and procalcitonin was associated with a 10.63, 2.87, 2.54, and 3.11% increase in the GM of IgG concentration, respectively. Any 10% increase in LDH, CRP, and ferritin was similarly associated with an 11.28, 2.48, and 3.0% increase in GM of IgG concentration at 6 months post-infection.

**Conclusion:**

Several clinical biomarkers in the acute phase of SARS-CoV-2 infection are associated with enhanced IgG antibody response detected after 6 months of disease onset. The measurement of SARS-CoV-2 specific antibody responses requires improved techniques and is not feasible in all settings. Baseline clinical biomarkers can be a useful alternative as they can predict antibody response during the convalescence period. Individuals with an increased level of NLR, CRP, LDH, ferritin, and procalcitonin may benefit from the boosting effect of vaccines. Further analyses will determine whether biochemical parameters can predict RBD-specific IgG antibody responses at later time points and the association of neutralizing antibody responses.

## Introduction

COVID-19 is an acute inflammatory disease, and the severity of infection is related to dysregulation of the inflammatory immune response (1). The disease caused by severe acute respiratory syndrome coronavirus 2 (SARS-CoV-2) has resulted in a critical threat to global health since the outbreak in December 2019 in China. Evidence suggests that patients with advanced age, respiratory distress, oxygen saturation of <90%, or pre-existing comorbidities are more susceptible to suffering from a severe disease (2, 3). Common clinical manifestations of COVID-19 patients include fever, cough, breathlessness, myalgia, fatigue, normal or decreased leukocyte counts, and radiographic evidence of pneumonia (4). A lack of immunity to the virus triggers the pathogenesis of the disease. Increased levels of inflammatory cells and markers in the blood, as well as high serum levels of several cytokines and chemokines, have been reported to be associated with increased disease severity and death (1). Higher levels of inflammatory markers (C-reactive protein and ferritin), liver enzymes, lactate dehydrogenase (LDH), D-dimer, creatine phosphokinase (CPK), and elevated inflammatory cytokines (IL-6, TNF-α) have been related with worse clinical outcome (5). Another study highlights the fact that the early formation of IgM and IgG antibodies does not necessarily lead to early elimination of SARS-CoV-2; however, the titer and specificity of antibodies may play a more important role in virus eradication (6).

Upon the entry of the virus into the cells, its antigen is presented to the host antigen presenting cells (APC) that play the central role in the body's antiviral immunity system. Antigen presentation subsequently stimulates the body's humoral and cellular immunity, which are mediated by virus-specific B and T cells. The SARS-specific IgM antibodies disappear at the end of week 12, while the IgG antibody can last for a long time, which may indicate that there is a protective role of IgG antibody (5). A community-based study showed that the kinetic of SARS-CoV-2-specific IgG antibody levels correlate with clinical parameters such as length and severity of infection (7). A trend of increasing antibody levels from asymptomatic to mild, moderate, and severe infection was observed from the study analysis (7). Although most predictive models rely on demographic features and data on clinical parameters obtained during hospitalization, time-dependent biomarkers, such as antibody titers and clinical laboratory values, substantially contributed to the development of a more accurate prediction model associated with COVID-19 severity and mortality (8).

Previous analysis has identified several clinical biomarkers, such as neutrophil-lymphocyte ratio (NLR), LDH, ferritin, C-reactive protein (CRP), and D-dimer, predictive for disease progression (2, 9, 10). In this analysis, we aimed to explore whether similar clinical biomarkers during the acute phase of SAR-CoV-2 infection help to predict long-term SARS-CoV-2-specific IgG antibody response post-infection.

## Methods

### Study participants and study sites

We enrolled 100 patients between November 2020 and February 2021 in Dhaka, Bangladesh, aged 18 years and above, who were confirmed positive using SARS-CoV-2 reverse transcription polymerase chain reaction (RT-PCR) for the first time prior to or during enrollment in a prospective cohort study as mentioned previously (2). We used WHO guidelines for COVID-19 (clinical symptoms and oxygen saturation) for determining the severity of the patients, which were collected from the hospital records on admission or the patient's condition during enrollment (11). Patients who provided a confirmed history of previous SARS-CoV-2 infection were excluded. During the enrollment period, none of our participants were vaccinated as COVID-19 vaccination in Bangladesh was initiated only in February 2021. Biweekly phone calls and scheduled clinic visits were carried out to collect information on vaccination status, clinical symptoms, and re-infection. In this analysis, we included all COVID-19 patients who completed the 3-month and 6-month follow-ups following enrollment. We excluded patients who were re-infected with SARS-CoV-2, defined as RT-PCR positive, or received any dose of the COVID-19 vaccine during or prior to the follow-up visits as it may have an impact on the antibody response. We selected all hospitalized patients from two COVID-19 designated hospitals: Kurmitola General Hospital and Mugda Medical College and Hospital as well as non-hospitalized patients from the community in Dhaka city. Ethical approval of the study was taken from the Institutional Review Board of the International Center for Diarrhoeal Disease Research, Bangladesh (icddr,b) and also from the Directorate General of Health Services (DGHS) of Bangladesh. Written informed consent was obtained from all study participants.

### Clinical and laboratory data

Socio-demographic data, comorbidities, anthropometric measurements, and relevant clinical information were recorded during enrollment (Day 1). All patients were prospectively followed up on day 7, day 14, day 28, day 90 (month 3), and day 180 (month 6). We considered the date of first symptom appearance as the disease onset date for all symptomatic cases. In the case of asymptomatic patients, the date of probable exposure with any COVID-19 patient plus a 2-day incubation period (12) or a positive report of RT-PCR was considered as the disease onset date.

We collected venous blood and nasopharyngeal swabs (NPS) from our study participants. NPS was collected for the detection of SARS-CoV-2 using CDC 2019-nCoV RT-PCR Diagnostic Panel (CDC 2019-nCoV_N2 primers and probe set) from China as mentioned earlier (2, 13). The clinical biomarkers consisting of complete blood count (CBC), alanine aminotransferase (ALT), CRP, LDH, ferritin, creatine phosphokinase (CK), procalcitonin, and D-dimer were measured on the day of enrollment for the study as per the national guideline for the management of COVID-19 (14). The ratio of absolute neutrophil count to absolute lymphocyte count was set as the NLR value (15). We measured the SARS-CoV-2-specific IgM and IgG responses to the receptor binding domain (RBD) as several groups showed a good correlation with levels of neutralizing antibody titers and COVID-19 severity (16–18).

### RBD-specific antibody responses from enzyme-linked immunosorbent assay (ELISA)

The RBD-specific antibody concentrations (ng/ml) were measured from blood samples collected at all follow-up visits by ELISA (isotype-specific anti-RBD monoclonal antibodies- Mab CR3022) (2, 19). We determine the cutoff for seropositivity as 500 ng/ml (0.5 μg/ml) for both IgG and IgM antibodies, which was the median plus the range of concentrations of SARS-CoV-2 IgG and IgM antibodies measured among pre-pandemic serum samples (19).

### Statistical analysis

Baseline characteristics were reported as mean with standard deviation (SD) for continuous variables and frequency measures for categorical variables. The distribution of clinical biomarkers during acute infection (day 1) was presented as a geometric mean (GM) with a 95% confidence interval (CI). Simple and multiple linear regression models were fitted to determine the association of the concentration of clinical biomarkers on enrollment (Day 1) with IgG concentration at 3 months and 6 months following enrollment. Both the outcome variables (IgG) and the predictors (clinical biomarkers) were log-transformed before putting into the models. We built separate models for each biomarker to avoid multicollinearity. All the multivariable models were adjusted for covariates of prior interest, such as age, sex, body mass index (BMI), blood group, diabetes mellitus, and the interval between disease onset and enrollment. These covariates were purposefully selected based on the literature review (20–22). The strength of association was reported as the percentage change in the GM of IgG (ng/ml) concentration for any 10% increase in the concentration of the clinical biomarkers (original unit). A *p*-value of <0.05 was considered a statistically significant association.

## Results

### Baseline characteristics of the cohort

**Figure 1:**
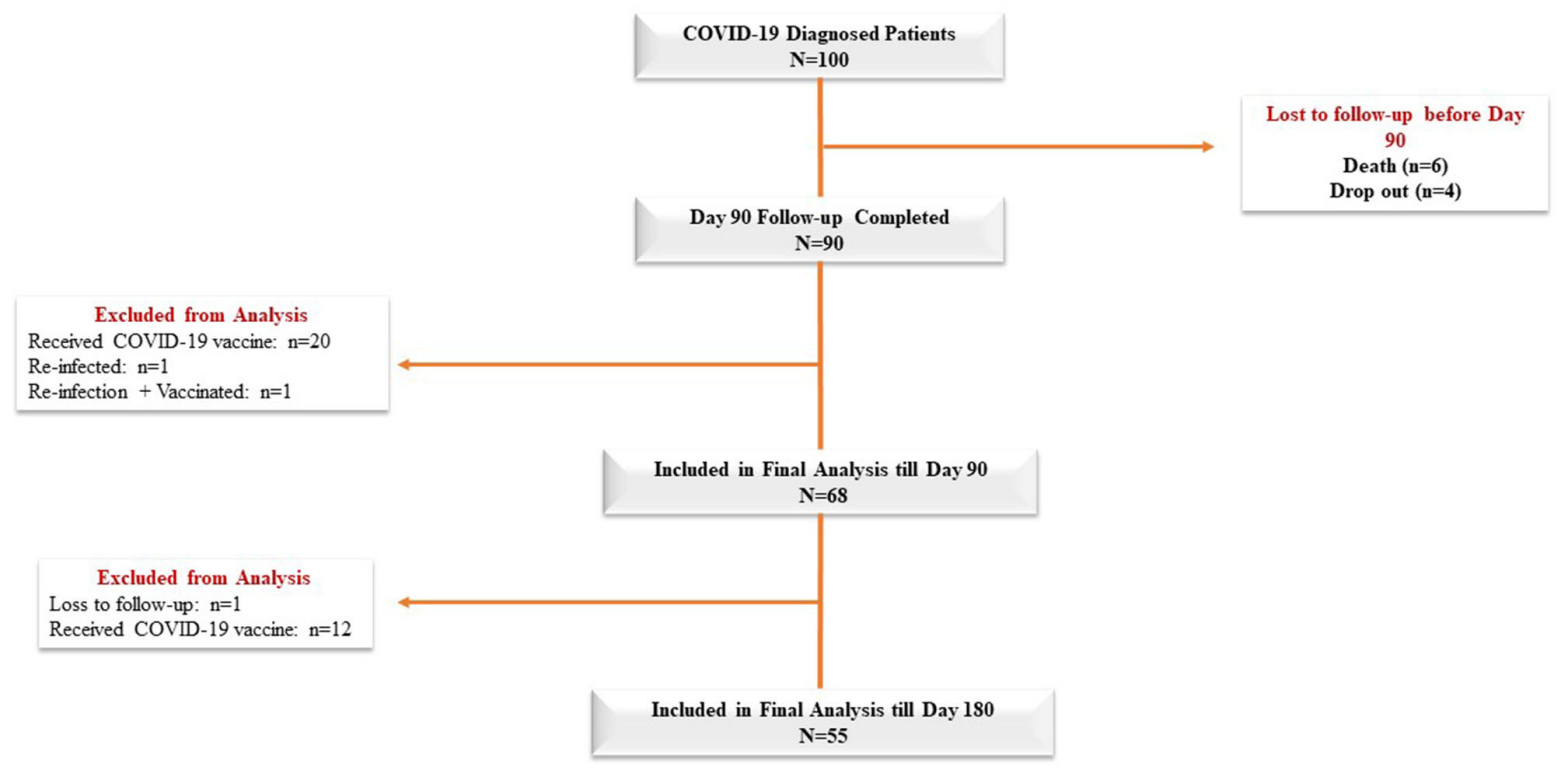
Analysis flow chart of cases.

The mean age of the 100 COVID-19 patients was 46.83 ± 14 years, and 60% of the patients in our cohort were male (Table 1). Diabetes mellitus was the most common comorbid disease (28%) in this cohort (Table 1). The mean BMI of the COVID-19 cases was 25.87 ± 3.65 kg/m^2^, and 32% of the patients belonged to the O blood group. In total, 68 and 55 COVID-19 patients were included in the final analysis who completed 3 months and 6 months of follow-up, respectively, after excluding re-infection, COVID-19 vaccines, death, and dropout cases (Figure 1). Patients were enrolled in the study at different time points from the disease onset, and the median days between symptom onset and enrolment were 10 days (IQR: 7.75, 12). The distribution of clinical biomarkers is outlined in Table 2. The geometric mean value of NLR 4.22 (95% CI: 3.40, 5.25), LDH 277.76 (95% CI: 251.56, 306.70), and CRP 1.45 (95% CI: 0.97, 2.17) was elevated above the normal range, whereas the geometric mean value of absolute lymphocyte counts 1.01(95% CI: 0.87, 1.17) was decreased during the acute infection (Table 2).

**Table 1:** Baseline characteristics of COVID-19 patients.

**Variables**	**All cases (*n =* 100)**	**COVID-19 patients at 3 months** **(*n =* 68)**	**COVID-19 patients at 6 months (*n =* 55)**
Age, (Mean, SD)	46.83 ± 14	45.0 ± 14.3	44.11 ± 14.86
Male, N (%)	60 (60)	40(58.8)	34 (61.8)
**Blood group, n (%)**			
A/AB	33 (33%)	23 (33.8)	19 (34.5)
B	35 (35%)	21 (30.9)	16 (29.1)
O	32 (32%)	24 (35.3)	20 (36.4)
BMI (kg/m^2^) (Mean ± SD)	25.87 ± 3.65	25.7 ± 3.42	25.8 ± 9
Interval between Disease Onset and enrolment, Median (IQR)^*^	10 (7.75, 12)	10 (7.5, 12)	9 (7.12)
**Comorbidities, n (%)**			
Liver Disease	1 (1)	1 (1.5)	1 (1.82)
Kidney Disease	4 (4)	3 (4.4)	2 (3.64)
Diabetes Mellitus	28 (28)	21 (30.9)	15 (27.3)

* For symptomatic COVID-19 cases, symptom onset date was considered as disease onset, whereas for asymptomatic cases, probable exposure date + incubation period (2 days) or the date of 1st COVID-19 positive was considered as disease onset.

**Table 2:** Baseline distribution of clinical biomarkers.

**Variables**	**Normal reference**	**GM, 95% CI**
Total leucocyte count (10^9^/L)	4.0–11.0	6.27 (5.72, 6.87)
Absolute neutrophil count (10^9^/L)	2.0–7.5	4.26 (3.76, 4.84)
Absolute lymphocyte count (10^9^/L)	1.5–4.0	1.01 (0.87, 1.17)
NLR	<4.795	4.22 (3.40, 5.25)
ALT (U/L)	<50	41.0 (33.85, 49.66)
LDH(U/L)	<248	277.76 (251.56, 306.70)
CRP (mg/dl)	<0.5	1.45 (0.97, 2.17)
Ferritin (ng/ml)	≤ 274.66	178.02 (121.10, 261.68)
Procalcitonin (ng/ml)	<0.1	0.07 (0.06, 0.09)
CPK (U/L)	<171	103.89 (81.38, 132.61)
D-Dimer (ng/ml)	<550	312.36 (261.95, 372.48)

### RBD-specific antibody responses

**Figure 2:**
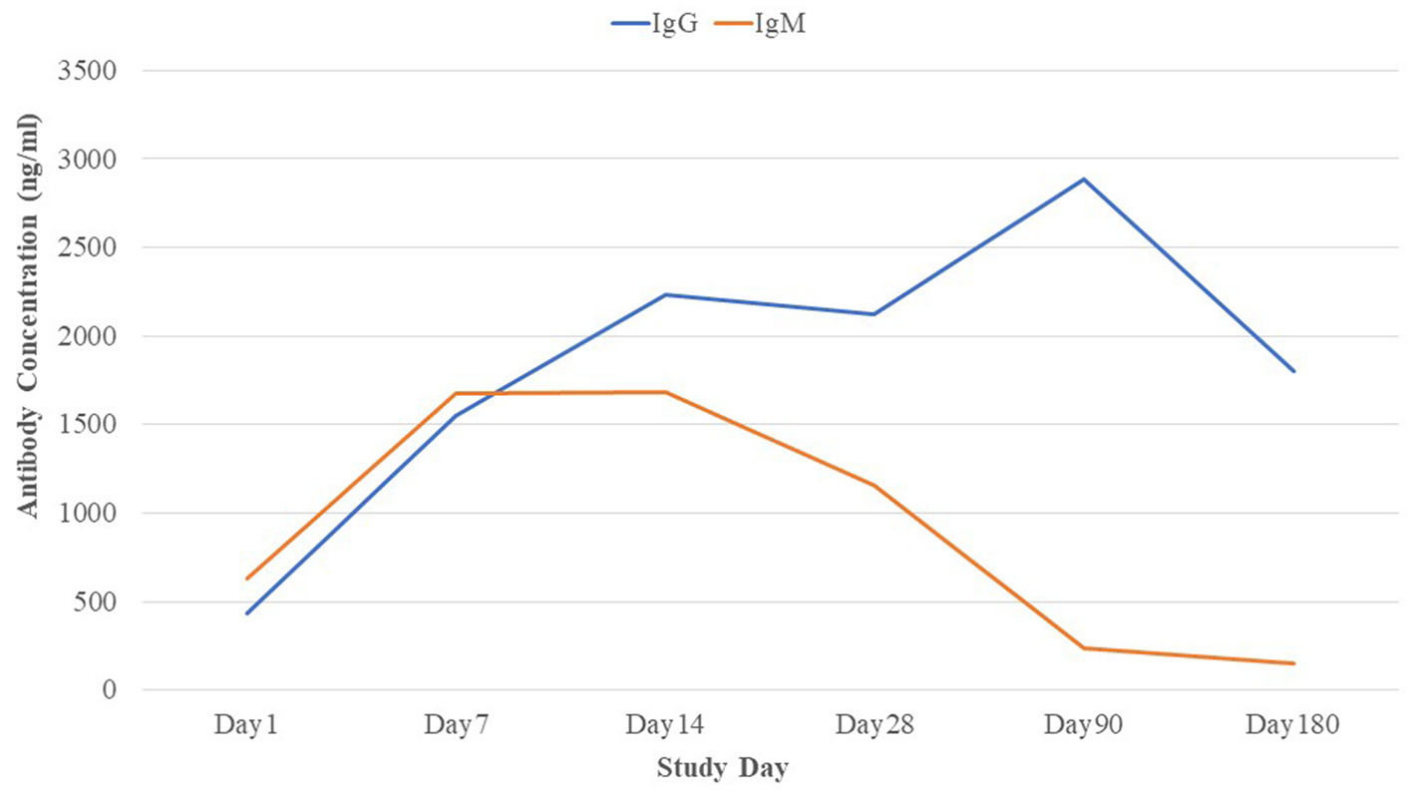
The trend of SARS-CoV-2-specific antibody IgG and IgM concentration over the time period.

**Figure 3:**
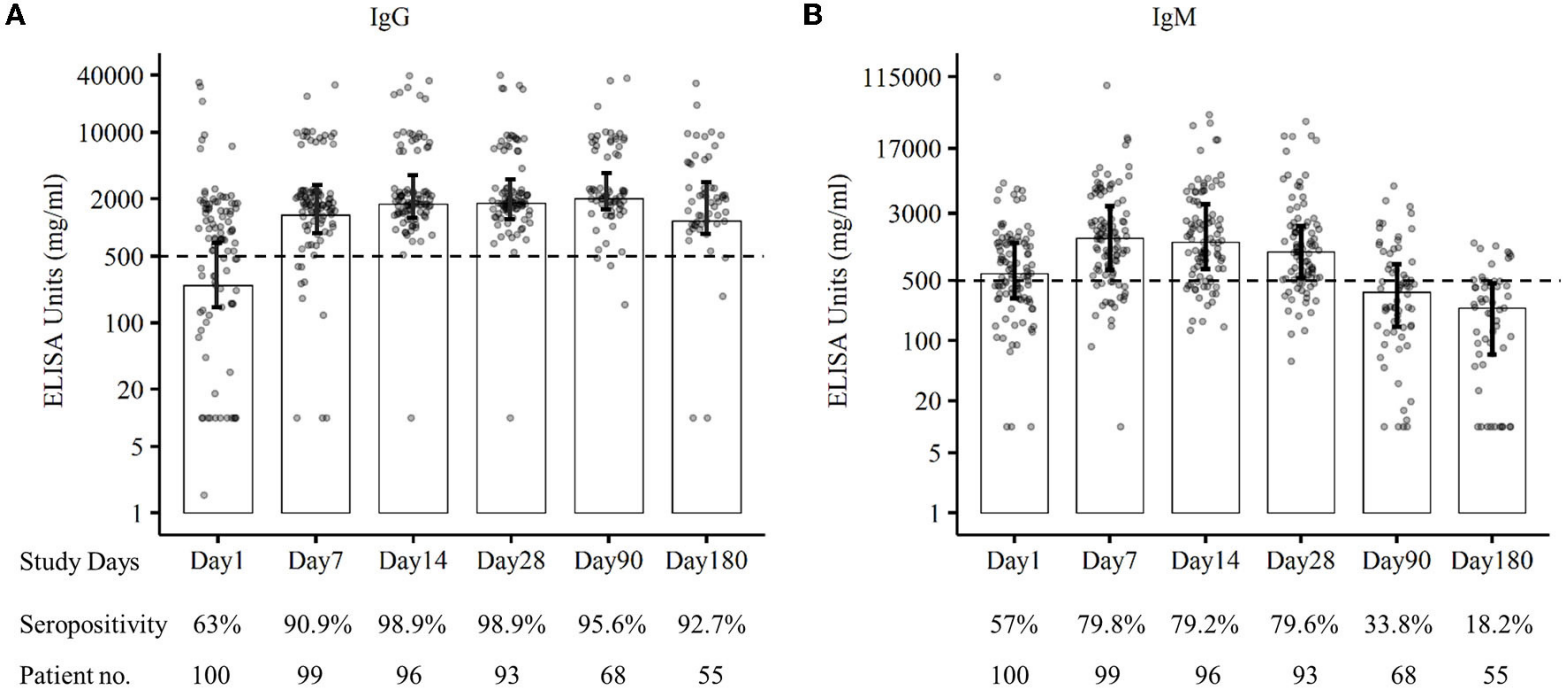
Seropositivity against SARS-CoV-2 RBD-specific IgG **(A)** and IgM **(B)** of patients at different follow-up days till 6 months.

The RBD-specific IgG responses increased initially till 3 months following the natural infection of COVID-19 and declined subsequently, whereas the RBD-specific IgM started declining within 1 month of infection after early responses (Figure 2). Approximately 96% of the patients were found seropositive against RBD-specific IgG at 3 months (day 90) and 93% of the patients were seropositive against RBD-specific IgG at 6 months (day 180) following infection (Figure 3A). Maximum (98.9%) IgG seropositivity was observed on day 14 and day 28. Only 33.8% of the patients were found seropositive against RBD-specific IgM at day 90, whereas only 18% of the patients had IgM concentration > 500 ng/ml at day 180 (Figure 3B). Maximum (79.8%) IgM seropositivity was observed on day 7.

### Clinical biomarkers predicting SARS-CoV-2 IgG response at 3 months post-infection

We found several clinical biomarkers during acute infection predicting SARS-CoV-2 RBD-specific IgG concentration during the convalescent period. In adjusted models, for any 10% increase in the hematological parameters, such as absolute lymphocyte count (10^9^/L) and NLR, there was a 5.32% (95% CI: 8.98, 1.53) decrease and 3.74% (95% CI: 0.93, 6.62) increase, respectively, in the GM of IgG (ng/ml). Any 10% increase in the inflammatory markers, such as LDH (U/L), CRP (mg/dl), ferritin (ng/ml), and procalcitonin (ng/ml), was associated with a 10.63% (95% CI: 4.33, 17.31), 2.87% (95% CI: 1.25, 4.51), 2.54% (95% CI: 0.59, 4.53), and 3.11% (95% CI: 0.32, 5.97) increase, respectively, in the GM of IgG antibody concentration (ng/ml). Absolute neutrophil count and D-dimer concentration on Day 1 were found to be significantly associated with IgG concentration at 3 months following infection in the unadjusted model, but the associations lost statistical significance after adjusting for the covariates (Table 3).

**Table 3:** Association of clinical biomarkers on day 1 with SARS-CoV-2 IgG at 3 months.

**Predictor variables**	**Unadjusted**		**Adjusted** ^*^	
	**PP (95% CI)** ^¶^	***P*** **-value**	**PP (95% CI)** ^¶^	***P*** **-value** ^#^
Total leucocyte count (10^9^/L)	1.05 (0.98, 1.12)	0.14	1.02 (0.95, 1.09)	0.56
Absolute neutrophil count (10^9^/L)	5.87 (1.27, 10.69)	0.013	3.37 (−1.81, 8.82)	0.20
Absolute lymphocyte count (10^9^/L)	−6.28 (9.68, −2.77)	0.001	−5.32 (−8.98, −1.53)	0.007
NLR	4.93 (2.43, 7.50)	<0.001	3.74 (0.93, 6.62)	0.010
ALT (U/L)	2.15 (−0.83, 5.22)	0.156	1.92 (−0.98, 4.91)	0.192
LDH (U/L)	11.63 (5.96, 17.61)	<0.001	10.63 (4.33, 17.31)	0.001
CRP (mg/dl)	3.13 (1.89, 4.39)	<0.001	2.87 (1.25, 4.51)	0.001
Ferritin (ng/ml)	2.35 (0.94, 3.77)	0.001	2.54 (0.59, 4.53)	0.011
Procalcitonin (ng/ml)	3.80 (1.13, 6.53)	0.006	3.11 (0.32, 5.97)	0.029
CPK (U/L)	0.67 (−1.68, 3.06)	0.576	0.72 (−2.06, 3.57)	0.611
D-Dimer (ng/ml)	4.37 (1.29, 7.55)	0.006	2.17 (−1.22, 5.68)	0.208

^*^Adjusted with covariates age, sex, BMI, blood group, diabetes mellitus, interval between disease onset, and enrollment. ^¶^Percentage change in geometric mean of IgG ng/ml for any 10% increase in clinical biomarkers (original unit). ^#^A *p*-value <0.05 was considered a statistically significant association.

### Clinical biomarkers predicting SARS-CoV-2 IgG response at 6 months post-infection

We found some common clinical biomarkers from the acute phase of infection predicting SARS-CoV-2 RBD-specific IgG concentration at 6 months post-infection (Table 4). In adjusted models, for any 10 % increase in LDH (U/L), CRP (mg/dl), and ferritin (ng/ml), there was 11.28% (95% CI: 4.19,18.85), 2.48% (95% CI: 0.49, 4.50), and 3.00% (0.59, 5.46) increase in the GM of IgG (ng/ml) at 6 months post-infection. Although procalcitonin and D-dimer were found significantly associated (*p* = 0.043 and *p* = 0.005, respectively) in the unadjusted model for predicting IgG concentration at 6 months, the associations lost statistical significance after adjusting with the covariates.

**Table 4:** Association of clinical biomarkers on day 1 with SARS-CoV-2 IgG at 6 months.

**Predictor variables**	**Unadjusted**		**Adjusted** ^*^	
	**PP (95% CI)** ^¶^	***P*** **-value**	**PP (95% CI)** ^¶^	***P*** **-value** ^#^
Total leucocyte count (10^9^/L)	0.03 (−8.76, 9.67)	0.994	0.41 (−9.91, 11.93)	0.939
Absolute neutrophil count (10^9^/L)	2.13 (−4.32, 9.01)	0.520	1.82 (−5.89, 10.16)	0.647
Absolute lymphocyte count (10^9^/L)	−4.97 (−10.60, 1.01)	0.100	−3.44 (−10.01, 3.61)	0.322
NLR	2.76 (−1.10, 6.77)	0.159	2.15 (−2.46, 6.97)	0.359
ALT (U/L)	2.81 (−0.83, 6.59)	0.128	2.81 (−0.83, 6.59)	0.171
LDH (U/L)	12.35 (6.13, 18.92)	<0.05	11.28 (4.19, 18.85)	0.002
CRP (mg/dl)	3.07 (1.55, 4.60)	<0.05	2.48 (0.49, 4.50)	0.015
Ferritin (ng/ml)	2.66 (0.93, 4.42)	0.003	3.00 (0.59, 5.46)	0.015
Procalcitonin (ng/ml)	3.22 (0.11, 6.43)	0.043	2.01 (−1.23, 5.36)	0.221
CPK (U/L)	0.73 (−1.98, 3.52)	0.596	0.56 (−2.58, 3.80)	0.725
D-Dimer (ng/ml)	4.79 (1.51, 8.18)	0.005	2.09 (−1.83, 6.16)	0.293

^*^Adjusted with covariates age, sex, BMI, blood group, diabetes mellitus, interval between disease onset and enrollment. ^¶^Percentage change in geometric mean of IgG ng/ml for any 10% increase in clinical biomarkers (original unit). ^#^A *p*-value <0.05 was considered a statistically significant association.

## Discussion

SARS-CoV-2 antibody responses are presently an important scientific issue as they can determine the seropositivity following infection and/or vaccination. The longevity of this immunity following SARS-CoV-2 infection may vary individually and depend on clinical parameters during infection (20). In this prospective study, we evaluated the clinical biomarkers (NLR, LDH, ferritin, CRP, procalcitonin, and D-dimer) during the acute phase of SAR-CoV-2 infection which will help to predict long-term SARS-CoV-2-specific IgG antibody response as late as 6 months following natural infection.

The immune responses after SARS-CoV-2 infection differ between patients suffering from varying ranges of severity (2). The reasons for the variation in immune responses to natural infection remain unclear and unpredictable. The factors responsible for long-term immunity after SARS-CoV-2 infection are also current topics of major scientific interest. IgG and IgM responses during the acute stage are well studied in different settings in the world, but the long-term immune responses are yet to be determined in South Asian countries like Bangladesh. Investigating the routine clinical biomarkers with long-term antibody responses in Bangladesh will provide an appropriate understanding of the protection of the disease. Such biomarkers may be a surrogate for determining long-term immune response in resource-limited settings where measuring antibody concentration is challenging.

We analyzed RBD-specific antibody responses of the COVID-19 patients following natural infection who completed 3 or 6 months of follow-up. We found that both the IgG and IgM responses commenced within 10 days of disease onset. Different studies showed that serum IgM and IgG responses were detectable due to the development of adaptive immunity within 5 to 7 days and 7 to 10 days, respectively, following the onset of symptoms (23–25). A similar trend of IgG and IgM responses was found in the primary analysis of this cohort in Bangladesh (2). In general, serum antibody level responses tend to decline after the acute phase of the disease as the “effector” response of B cells is stimulated during the first weeks after infection (25).

We also found that high levels of the IgM responses persisted for 1 month following disease onset and began to decline gradually. A similar finding was confirmed by the other study conducted in China (26). The IgM response became lower than the cutoff value after the 3rd month of SARS-CoV-2 infection. In our study, we observed a declining trend of SARS-CoV-2 RBD-specific IgG responses after 3 months of infection though 95.6% of patients were seropositive at the end of 3 months. The seropositivity was 92.7% during 6 months of follow-up, and similar sero-responses were found in the other study conducted in Germany (20). Some studies suggested that anti-SARS-CoV-2-specific IgG antibodies declined at 3 months following an infection (27, 28), whereas others reported IgG responses sustained over 3 to 6 months (26, 29, 30). However, our findings on the long-term trend of SARS-CoV-2 antibody response are the first from Bangladesh, which is consistent with the results observed elsewhere (20, 26–30). Further studies are necessary to find out a clearer understanding of the anti-SARS-CoV-2- specific IgG antibody responses.

COVID-19 is a multisystem disease instead of a localized respiratory infection, caused by a diffuse systemic process involving a complex interplay of immunological, inflammatory, and coagulative cascades (31). Excessive pro-inflammatory response of the former and dysregulated host response of the latter lead to tissue damage. The extensive immune dysregulation results in the release of massive amounts of cytokines and chemokines typically known as the “cytokine storm” (31), which is characterized by an increase in several pro-inflammatory cytokines in the serum concentrations. Thus, lymphocyte counts and cytokine concentrations could be used as biomarkers to predict the severity grading and persistence of the disease (5). In our earlier analysis, we observed that individuals suffering from moderate-to-severe COVID-19 had higher SARS-CoV-2 RBD-specific IgG concentration compared to the mild and asymptomatic cases indicating that immune responses are correlated with disease severity (2). It has been studied that the magnitude of early CD4+ T-cell immune responses correlates with the severity of initial infection, although there was no correlation between soluble inflammatory markers, such as D-dimer, and the long-term T-cell responses (21).

Following infection, several acute-phase reactants, such as CRP, LDH, ferritin, and D-dimer, play a crucial role in inflammatory responses (32). CRP and LDH are plasma proteins, which are induced by various inflammatory mediators and are clinically used as biomarkers for various inflammatory conditions (33). Similarly, increased serum ferritin levels during acute infection indicate a significant host defense mechanism by limiting the availability of iron to pathogens and protecting immune cell function (34). In our analysis, we found a strong statistical association between CRP, LDH, and ferritin in predicting long-term SARS-CoV-2-specific IgG concentration at both 3-month and 6-month periods following infection, suggesting that elevated levels of these biomarkers during acute infection may surrogate the presence of antibody response as late as 6 months post-infection. On the other hand, elevated serum procalcitonin was observed commonly in hospitalized COVID-19 patients ranging from moderate-to-severe forms of the disease (2, 35). Procalcitonin is also an acute-phase reactant and is usually been associated with bacterial infections (36). This biomarker was significantly associated with predicting IgG concentration at 3 months post-infection but not at a later time point.

The coagulative cascade involves endothelial cells, platelets, neutrophils, monocytes, and macrophages. The vascular endothelium of a healthy individual is both anti-thrombotic and anti-inflammatory, which is disrupted in COVID-19 leading to thrombosis and inflammation. Macrophages, recruited to fibrin thrombi, generate plasmin, through which fibrin is degraded to D-dimers. Thus, macrophages possibly contribute to the unusually extreme elevation of D-dimers seen in COVID-19 (31). However, in our analysis D-dimer was was not significantly associated in predicting IgG concentration at both 3 and 6 months post infection.

One of the major limitations of this analysis was the final sample size included in the analysis. The sample analyzed for the 3-month and 6-month models is different from the original sample of 100 patients (2) because of the loss to follow-up, including death, and exclusion of cases with re-infection and vaccination. However, the background characteristics remain quite comparable between the samples. As the original cohort enrolled patients purposely based on severity, we were unable to adjust for disease severity due to bias. In the case of asymptomatic patients, as there were no symptoms, we assumed the disease onset was based on assumptions of the date of exposure with COVID-19 patient or RT-PCR positive report.

## Conclusion

In this current analysis, we were able to identify some of the routinely performed clinical biomarkers during acute infection in predicting SARS-CoV-2 IgG responses over the time period, which suggested the importance of longevity of the immune response. The measurement of SARS-CoV-2-specific antibody responses is not feasible in all settings as it requires improved techniques. In Bangladesh, measuring RBD-specific IgM and IgG is approved by the government only for institutional and research purposes; hence, it is not easily available in hospitals or clinics. Thus, routinely performed clinical biomarkers during acute infection will serve as surrogate markers in predicting long-term immune response as late as 6 months post-infection. Additionally, patients exhibiting increased levels of NLR, CRP, LDH, ferritin, and procalcitonin during acute infection may benefit from boosting effect on the SARS-CoV-2 IgG concentration even with a single dose of COVID-19 vaccine after 3 months of infection (37). Although in our study we only analyzed the RBD-specific antibody response, previous studies have shown that RBD-specific antibody concentration correlates with neutralizing antibody titers (16, 17, 38). In addition, several studies have also shown a correlation between RBD-specific antibody titer and disease severity (2, 8, 18). However, to better understand the correlates of protection, a prospective analysis will be needed to study the association of neutralizing antibody with the acute-phase reactant biomarkers and RBD-specific antibody response.

## Data availability statement

The raw data supporting the conclusions of this article will be made available by the authors, without undue reservation.

## Ethics statement

The studies involving human participants were reviewed and approved by Institutional Review Board (IRB) of the International Centre for Diarrhoeal Disease Research (icddr,b). The patients/participants provided their written informed consent to participate in this study.

## Author contributions

TasA, AA, IT, TB, FC, and FQ designed and supervised the study. TasA, AA, IT, MA, and SR helped to collect the specimens and performed laboratory work and immunological analyses. TasA and SH analyzed the data and drafted the manuscript. TasA, SH, AA, IT, MA, SR, TB, TahA, FQ, and FC reviewed the manuscript. All authors contributed to the interpretation of results and critical review and revision of the manuscript and have approved the final version.

## References

[1] García LF. Immune response, inflammation, and the clinical spectrum of COVID-19. Front Immunol. (2020) 11:1441. 10.3389/fimmu.2020.01441 32612615 PMC7308593

[2] Akter A Ahmed T Tauheed I Akhtar M Rahman SIA Khaton F. Disease characteristics and serological responses in patients with differing severity of COVID-19 infection: a longitudinal cohort study in Dhaka, Bangladesh. PLoS Negl Trop Dis. (2022) 16:e0010102. 10.1371/journal.pntd.0010102 34982773 PMC8759637

[3] Marin BG Aghagoli G Lavine K Yang L Siff EJ Chiang SS. Predictors of COVID-19 severity: a literature review. Rev Med Virol. (2021) 31:e2146. 32845042 10.1002/rmv.2146 PMC7855377

[4] Li X Geng M Peng Y Meng L Lu S. Molecular immune pathogenesis and diagnosis of COVID-19. J Pharm Anal. (2020) 10:102–8. 10.1016/j.jpha.2020.03.001 32282863 PMC7104082

[5] Papadopoulou G Manoloudi E Repousi N Skoura L Hurst T Karamitros T. Molecular and clinical prognostic biomarkers of COVID-19 severity and persistence. Pathogens. (2022) 11:311. 10.3390/pathogens11030311 35335635 PMC8948624

[6] Wang X Huang K Jiang H Hua L Yu W Ding D. Long-Term existence of SARS-CoV-2 in COVID-19 patients: host immunity, viral virulence, and transmissibility. Virol Sin. (2020) 35:793–802. 10.1007/s12250-020-00308-0 33156486 PMC7644793

[7] Sasisekharan V Pentakota N Jayaraman A Tharakaraman K Wogan GN Narayanasami U. Orthogonal immunoassays for IgG antibodies to SARS-CoV-2 antigens reveal that immune response lasts beyond 4 mo post illness onset. Proc Natl Acad Sci. (2021) 118:e2021615118. 10.1073/pnas.2021615118 33446512 PMC7865175

[8] Lasso G Khan S Allen SA Mariano M Florez C Orner EP. Longitudinally monitored immune biomarkers predict the timing of COVID-19 outcomes. PLOS Comput Biol. (2022) 18:e1009778. 10.1371/journal.pcbi.1009778 35041647 PMC8812869

[9] Moutchia J Pokharel P Kerri A McGaw K Uchai S Nji M. Clinical laboratory parameters associated with severe or critical novel coronavirus disease 2019 (COVID-19): a systematic review and meta-analysis. PLoS One. (2020) 15:e0239802. 10.1371/journal.pone.0239802 33002041 PMC7529271

[10] Lin Z Long F Yang Y Chen X Xu L Yang M. Serum ferritin as an independent risk factor for severity in COVID-19 patients. J Infect. (2020) 81:647–79. 10.1016/j.jinf.2020.06.053 32592705 PMC7313486

[11] World Health Organization. Clinical management of COVID-19: Interim Guidance, 27 May 2020. Geneva: World Health Organization (2020

[12] Backer JA Klinkenberg D Wallinga J. Incubation period of 2019 novel coronavirus (2019-nCoV) infections among travellers from Wuhan, China, 20–28 January 2020. Eurosurveillance. (2020) 25:2000062. 10.2807/1560-7917.ES.2020.25.5.2000062 32046819 PMC7014672

[13] CDC. Research Use Only 2019-Novel Coronavirus (2019-nCoV) Real-time RT-PCR Primers and Probes. Cent Dis Control Prev. (2020) Available online at: https://www.cdc.gov/coronavirus/2019-ncov/lab/rt-pcr-panel-primer-probes.html (Accessed April 25, 2021).

[14] National Guidelines on Clinical Management of COVID-19 version 8.0. (2021) Available online at: https://covidlawlab.org/wp-content/uploads/2021/01/Bangladesh_2020.11.05_Guideline_National-Guidelines-on-Clinical-Management-of-COVID-19_EN.pdf (accessed August 23, 2021).

[15] Wang Y Zhao J Yang L Hu J Yao Y. Value of the neutrophil-lymphocyte ratio in predicting COVID-19 severity: a meta-analysis. Dis Markers. (2021) 2021:2571912. 10.1155/2021/2571912 34650648 PMC8510823

[16] Poon RW-S Lu L Fong CH-Y Ip T-C Chen L-L Zhang RR-Q. Correlation between commercial Anti-RBD IgG titer and neutralization titer against SARS-CoV-2 Beta Variant. Diagnostics. (2021) 11:2216. 10.3390/diagnostics11122216 34943453 PMC8700542

[17] Padoan A Bonfante F Cosma C Chiara CD Sciacovelli L Pagliari M. Analytical and clinical performances of a SARS-CoV-2 S-RBD IgG assay: comparison with neutralization titers. Clin Chem Lab Med CCLM. (2021) 59:1444–52. 10.1515/cclm-2021-0313 33855843

[18] Gozalbo-Rovira R Gimenez E Latorre V Francés-Gómez C Albert E Buesa J. SARS-CoV-2 antibodies, serum inflammatory biomarkers and clinical severity of hospitalized COVID-19 patients. J Clin Virol Off Publ Pan Am Soc Clin Virol. (2020) 131:104611. 10.1016/j.jcv.2020.104611 32882666 PMC7459327

[19] Shirin T Bhuiyan TR Charles RC Amin S Bhuiyan I Kawser Z. Antibody responses after COVID-19 infection in patients who are mildly symptomatic or asymptomatic in Bangladesh. Int J Infect Dis. (2020) 101:220–5. 10.1016/j.ijid.2020.09.1484 33031941 PMC7534791

[20] Gerhards C Thiaucourt M Kittel M Becker C Ast V Hetjens M. Longitudinal assessment of anti-SARS-CoV-2 antibody dynamics and clinical features following convalescence from a COVID-19 infection. Int J Infect Dis. (2021) 107:221–7. 10.1016/j.ijid.2021.04.080 33932604 PMC8080496

[21] Peluso MJ Deitchman AN Torres L Iyer NS Munter SE Nixon CC. Long-term SARS-CoV-2-specific immune and inflammatory responses in individuals recovering from COVID-19 with and without post-acute symptoms. Cell Rep. (2021) 36:109518. 10.1016/j.celrep.2021.109518 34358460 PMC8342976

[22] Zhou Y Chi J Lv W Wang Y. Obesity and diabetes as high-risk factors for severe coronavirus disease 2019 (Covid-19). Diabetes Metab Res Rev. (2021) 37:e3377. 10.1002/dmrr.3377 32588943 PMC7361201

[23] Long Q-X Liu B-Z Deng H-J Wu G-C Deng K Chen Y-K. Antibody responses to SARS-CoV-2 in patients with COVID-19. Nat Med. (2020) 26:845–8. 10.1038/s41591-020-0897-1 32350462

[24] Ng DL Goldgof GM Shy BR Levine AG Balcerek J Bapat SP. SARS-CoV-2 seroprevalence and neutralizing activity in donor and patient blood. Nat Commun. (2020) 11:4698. 10.1038/s41467-020-18468-8 32943630 PMC7499171

[25] Stephens DS McElrath MJ. COVID-19 and the Path to Immunity. JAMA. (2020) 324:1279–81. 10.1001/jama.2020.16656 32915201 PMC12177933

[26] Liu C Yu X Gao C Zhang L Zhai H Hu Y. Characterization of antibody responses to SARS-CoV-2 in convalescent COVID-19 patients. J Med Virol. (2021) 93:2227–33. 10.1002/jmv.26646 33135795

[27] Ibarrondo FJ Fulcher JA Goodman-Meza D Elliott J Hofmann C Hausner MA. Rapid Decay of Anti–SARS-CoV-2 Antibodies in Persons with Mild Covid-19. N Engl J Med. (2020) 383:1085–7. 10.1056/NEJMc2025179 32706954 PMC7397184

[28] Röltgen K Powell AE Wirz OF Stevens BA Hogan CA Najeeb J. Defining the features and duration of antibody responses to SARS-CoV-2 infection associated with disease severity and outcome. Sci Immunol. (2020) 5:eabe0240. 10.1126/sciimmunol.abe0240 33288645 PMC7857392

[29] Tian X Liu L Jiang W Zhang H Liu W Li J. Potent and persistent antibody response in COVID-19 recovered patients. Front Immunol. (2021) 12:659041. 10.3389/fimmu.2021.659041 34122416 PMC8193946

[30] Wang K Long Q-X Deng H-J Hu J Gao Q-Z Zhang G-J. Longitudinal dynamics of the neutralizing antibody response to severe acute respiratory syndrome coronavirus 2 (SARS-CoV-2) Infection. Clin Infect Dis. (2021) 73:e531–9. 10.1093/cid/ciaa1143 32745196 PMC7454328

[31] Samprathi M Jayashree M. Biomarkers in COVID-19: an up-to-date review. Front Pediatr. (2021) 8:607647. 10.3389/fped.2020.607647 33859967 PMC8042162

[32] Chan AS Rout A. Use of neutrophil-to-lymphocyte and platelet-to-lymphocyte ratios in COVID-19. J Clin Med Res. (2020) 12:448–53. 10.14740/jocmr4240 32655740 PMC7331861

[33] Kermali M Khalsa RK Pillai K Ismail Z Harky A. The role of biomarkers in diagnosis of COVID-19 – A systematic review. Life Sci. (2020) 254:117788. 10.1016/j.lfs.2020.117788 32475810 PMC7219356

[34] Kernan KF Carcillo JA. Hyperferritinemia and inflammation. Int Immunol. (2017) 29:401–9. 10.1093/intimm/dxx031 28541437 PMC5890889

[35] Ticinesi A Nouvenne A Prati B Guida L Parise A Cerundolo N. The clinical significance of procalcitonin elevation in patients over 75 years old admitted for COVID-19 pneumonia. Mediators Inflamm. (2021) 2021:5593806. 10.1155/2021/5593806 34326704 PMC8245241

[36] Hussain A Singh L Iii JM Jo Y Makaryan TT Hussain S. Serum procalcitonin as a predictive biomarker in COVID-19: a retrospective cohort analysis. Cureus. (2022) 14:27816. 10.7759/cureus.27816 36106293 PMC9452059

[37] CDC Guideline on COVID-19 Immunization Schedule. (2020) Available online at: https://www.cdc.gov/vaccines/covid-19/downloads/COVID-19-immunization-schedule-ages-6months-older.pdf (Accessed November 20, 2022).

[38] Wagner A Guzek A Ruff J Jasinska J Scheikl U Zwazl I. Neutralising SARS-CoV-2 RBD-specific antibodies persist for at least six months independently of symptoms in adults. Commun Med. (2021) 1:1–11. 10.1038/s43856-021-00012-4 35602189 PMC9037317

